# CT Colonography and Colorectal Carcinoma: Current Trends and Emerging Developments

**DOI:** 10.7759/cureus.24916

**Published:** 2022-05-11

**Authors:** Monika Kadari, Muhammad Subhan, Nisha Saji Parel, Parimi Vamsi Krishna, Anuradha Gupta, Kamsika Uthayaseelan, Kivonika Uthayaseelan, Naga Anjani Bhaskar Srinivas Sunkara

**Affiliations:** 1 Internal Medicine, Bhaskar Medical College, Hyderabad, IND; 2 Internal Medicine, Allama Iqbal Medical College, Jinnah Hospital, Lahore, PAK; 3 Family Medicine, Tbilisi State Medical University, Tbilisi, GEO; 4 Internal Medicine, Jagadguru Jayadeva Murugarajendra Medical College, Davangere, IND; 5 Research, Government Medical College Nagpur, Nagpur, IND; 6 Internal Medicine, All Saints University College of Medicine, St. Vincent & the Grenadines, Kingstown, VCT; 7 Internal Medicine, All Saints University School of Medicine, Dominica, Roseau, DMA

**Keywords:** invasive colon cancer, colo rectal cancer, fobt, flexible sigmoidoscopy, barium enema, colorectal polyp, spiral ct, ct colonography, screening colonoscopy, colon cancer prevention

## Abstract

Colorectal carcinoma is the third most malignant and second leading cause of death from cancer. The cruelty of this entity is that it takes decades to be symptomatic and is known to be detected late in its timeline by a screening technique. The fatality of this carcinoma only means heightened importance of screening guidelines to be laid down and strict follow-ups by the healthcare providers. A novel method, a potential competitor that could now replace the present screening techniques for colorectal carcinoma, is computed tomographic colonography (CTC) or virtual colonoscopy. Though it first came into existence in 1994, this method is yet to be deeply studied and scrutinized for it to be the next benchmark modality. This review has mainly focused on the various features of CTC. It is contrasted against the gold standard colonoscopy for its superiority, efficacy, cost-effectiveness, patient logistics, and role in detecting extra-colonic lesions. The main focus would be laid on CTC being a screening modality. The review also emphasized why there is a need for the current healthcare providers to incorporate this modality into their practice widely. Although much has been said about CTC and its various aspects of cost-effectiveness, about it being replaced or supplemented for cancer screening, a collaborative effort has to be made by both the fields of radiology and gastroenterology to investigate the outcomes of this not so new technique in daily practice and to avoid misinterpretation of the results due to lack of skill and proficiency.

## Introduction and background

Most colon cancers form over time due to a succession of histological, morphological, and genetic changes [1]. This has enabled the diagnosis and screening of precancerous polyps in patients at average risk of colorectal cancer (CRC) before they turn cancerous. The capacity of screening to avoid cancer morbidity, mortality, and high treatment costs by discovering large lesions before they turn malignant, and early-stage cancer before it spreads beyond the intestinal wall demonstrates its clinical usefulness [1]. CRC takes consolation as the third most commonly occurring carcinoma and the second leading cause of death from cancer [2]. The majority of CRC instances are found in Western countries, and the disease's prevalence is increasing year by year [3].

Approximately 60%-70% of confirmed cases in symptomatic patients are discovered at an advanced stage of disease at this time [4]. The incidence of CRC varies worldwide, with developed countries having greater rates than developing countries [5]. Low socioeconomic status is linked to an increased risk of CRC and inadequate risk behavior and medical care. The lifetime average incidence of CRC in White Americans is 5% [5]. However, it is higher in males than women. The bulk of CRCs is carcinomas, with adenocarcinomas and other uncommon cancers accounting for more than 90% of all cases (adenosquamous, spindle, squamous, and undifferentiated). Medullary, cribriform comedo-type, micropapillary, serrated, mucinous, and signet-ring cell adenocarcinomas are the different types of CRC adenocarcinomas [5]. Men are more affected than women to acquire CRC, and their risk rises with age, especially after 50 years [4]. Epidemiological studies show that CRC is strongly linked to the environment and lifestyle [5]. Obesity, red meat, tobacco smoking, consumption of alcohol, androgen deprivation therapy, and cholecystectomy are all linked to a slight increase in CRC risk. Large population studies with varying degrees of evidence have discovered CRC protective factors such as exercise, a diet abundant with fruits and vegetables, rich in fiber, complex starch, fish, vitamin supplementation with folic acid, pyridoxine B6, calcium, vitamin D, magnesium, caffeine, and drugs like aspirin, non-steroidal anti-inflammatory drugs (NSAIDs), hormonal replacement therapy in postmenopausal women, statins, bisphosphonates, and angiotensin [5].

According to clinical evidence, CRCs are commonly caused by adenomatous polyps, which typically develop dysplastic alterations in a decade before developing invasive carcinoma [5]. Early diagnosis and excision of polyps can minimize the risk of CRC. Chromosomal instability, mismatch repair, and hypermethylation are three key molecular mechanisms implicated in CRC. Hypermethylation of 5'-cytosine-phosphate-guanine-3' (CpG) sites in deoxyribonucleic acid (DNA) can either activate or suppress the expression of specific genes, such as B-Raf and V-Raf murine sarcoma viral oncogene homolog B (BRAF) and MutL1 homolog 1 (MLH1) [5]. Somatic mutations in oncogenes (rat sarcoma virus, Ras; Src proto-oncogene, non-receptor tyrosine kinase, Src; Myc proto-oncogene, Myc) have been linked to CRC, with Ras having the highest clinical significance [5].

CRC grows slowly and does not usually cause symptoms until it has grown to a size of several centimeters, at which point it can impede the passage of feces and cause cramping, pain, or bleeding, which can appear as visible blood with bowel movements or, more infrequently, dark "tarry" stools [1]. Computed tomographic colonography (CTC) is similar to colonoscopy (OC) but less invasive. OC sensitivity ranges from 94.7% (95% confidence interval [CI] 90% to 97%) and can miss anywhere from 2% to 6% of cases, primarily on the right side, depending on preparation quality and hands-on competence [5]. When both cathartic and tagging agents are used in bowel preparation, CTC is extremely sensitive for CRC [6]. Given the low frequency of CRC, primary CTC, assuming appropriate specificity, may be preferable to OC for the initial evaluation of suspected CRC [6]. The only instrument that can evaluate the entire colon and detect and eliminate precancerous tumors is OC [4]. It is used extensively as a primary screening test or diagnostic test when another primary screening modality has yielded a positive result. It is worth noting that OC comes with many potential side effects, both from the inspection and conscious sedation or anesthetic used during the operation [4]. The invasiveness of OC is a major drawback for screening, and a thorough assessment of the patient's comorbidities and the risk-benefit ratio is always required [7]. CTC is one of the newest techniques for colon research, particularly in Western countries [4]. It exposes patients to minimal radiation levels while avoiding the hazards of intubation and sedation, and it is a feasible technique for patients who cannot or will not endure OC or sedation. The aim is to analyze, compare and dissect the cost-effectiveness, efficacy, superiority of CTC versus OC screening [6]. Virtual colonoscopy (VC), commonly known as CT colonography, is a minimally invasive imaging examination of the large intestine [8]. In essence, CTC is a modified CT scan in which the pictures are analyzed utilizing modern 2D, and 3D presentation techniques after the patient has undergone bowel preparation and colonic distention. The roots of CTC as a natural extension of abdominal CT imaging will be covered in this review, along with the evolution of CTC through the clinical phases of feasibility, validation, and implementation [8].

## Review

Current CRC screening guidelines

CRC being the third commonest cancer in the world and the second most common cause for cancer-related deaths with over 880,000 in 2018 is indeed deadly [2]. Between 2008 and 2012, the average yearly changes in the early-onset CRC incidence in New Zealand were 4.0%, 2.8% in Canada and Australia, and 2.2% in the United States [9]. Since the mid-1990s, the incidence of early-onset CRC has been rising in the United States [10]. Also, the incidence of early-onset CRC per 100,000 people increased from 5.9 cases in 2000 to 8.4 cases in 2017 [11]. Although total CRC incidence rates in many high-income nations have remained consistent or decreased, the incidence of early-onset CRC (i.e., diagnosis of CRC before age 50) has lately tremendously increased [9,12]. Rectal cancer was initially the leading cause of early-onset CRC in the United States. Rectal cancer incidence increased by 3.2% for those aged 20-39 between 1980 and 2013, and by 2.3% for those aged 40-49 between 1991 and 2013, whereas the incidence of colon cancer increased by 2.4% for those aged 20-29, 1.0% for those aged 30-39 between 1988 and 2013, and 1.3% for those aged 40-49 between 1996 and 2013 [10].

CRC mortality can be reduced through screening and early detection, and cancer incidence can be reduced by removing neoplastic lesions [13]. While research into the risk factors, etiology, and precursor lesions of CRC has progressed, the explanation for the current rise in cancer among young adults remains unexplained [13]. With the implementation of national screening programs, the early detection of adenomas and small tumors during OC has significantly improved, lowering the incidence of CRC [14]. Since its inception in 2002, this has been reduced by 17%-26% in Germany.

In a study performed in 2006 by Lin et al., CRC was found in 13.8% of those aged 50-54, 26.5% of those aged 75-79, and 28.6% of those aged 80 and up [15]. The mean increase in life expectancy was substantially smaller in the 80-year-old group compared to the 50- to 54-year-old group (0.13 vs. 0.85 years). However, there was a more significant prevalence of CRC in senior patients [15]. It was observed that with longer polyp latency, the elderly had a substantially lower addition in life expectancy than the younger patients in a sensitivity analysis; conversely, if it was presumed that a small subset of adenomas advances to CRC, the elderly had a lower average renewal in life expectancy than the younger patients. They arrived at a conclusion of only a 15% increase in life expectancy as shown in the study after screening >85-year-old age group, implying that risks and benefits should be carefully considered in many older people [15].

In a detailed study conducted by Sehgal et al., at the age of 45-49 years, 276 and 4844 people developed CRC, respectively, out of 195,600 people who had an OC and 2.6 million who did not, resulting in CRC incidence rates of 20.8 (95% CI 18.5-23.4) and 30.6 (95% CI 29.8-31.5) per 100,000 person-years [16]. At the age of 50-54 years, 798 and 6757 people out of 660,248 with and 2.4 million without OC developed CRC, resulting in CRC incidence rates per 100,000 person-years of 51.9 (95% CI 0.7-53.1) and 19.0 (95% CI 17.7-20.4), respectively. The hazard ratios that were adjusted for incident CRC after OC for ages 45-48 years were 0.50 (95% CI 0.44-0.56) and for ages 50-54 years 0.32 (95% CI 0.29-0.34) [16]. The findings were comparable for both women and men (0.48 hazard ratio, 95% CI 0.40-0.57; 0.52 hazard ratio, 95% CI 0.43-0.62, at ages 45-49 years; 0.35 hazard ratio, 95% CI 0.31-0.39; hazard ratio 0.29, 95% CI 0.26-0.32, at ages 50-54 years, respectively). This study concluded that a significant reduction in the CRC incidence later in life is observed when OC is done between the ages of 45 and 49, or between the ages of 50 and 54, linked to having a potential influence on screening recommendations.

Adults should be screened for CRC starting at the age of 50 and continuing until they are 75 years old, according to the US Preventive Services Task Force (USPSTF) [17]. In this subset of patients, screening has resulted in a lower incidence of CRC. Amidst these breakthroughs, persons aged 20-49 years and those older than 75 years show an increased incidence of CRC [17]. Because of the high prevalence and mortality of CRC, screening programs for this disease must be constantly improved to reduce its incidence [18]. The average risked individuals between ages 50 and 75 must be screened [19]. Recommendations for optimal surveillance intervals, preferred tests/test cascades, and the best time to start and stop screening vary by region and should be considered when making clinical decisions. Furthermore, the availability of local resources and patient preferences are essential in increasing the CRC screening uptake, as any screening is preferable to none [19].

Microsimulation Screening Analysis (MISCAN) and Simulation Model of Colorectal Cancer (SimCRC) were two methods first employed in the United States. In 2009, a study recommended that screening should begin at the age of 50 with annual fecal occult blood testing (FOBt), OC done every 10 years, or FOBt every two to three years plus flexible sigmoidoscopy (FS) every five years [20]. It looked into the possibility of starting screening earlier (at 45 years) with a longer screening interval (OC every 15 years), but the researchers concluded that the small number of life-years gained was not justified given the lack of evidence to support earlier screening; furthermore, starting screening at 50 years of age with the yearly fecal immunochemical testing (FIT) with OC done every 10 years, FS along with annual FIT every 10 years, or OC done every five years balanced the risks and benefits against the number of life-years gained as shown in Table 1 [21].

**Table 1:** USPSTF screening guidelines USPSTF, US Preventive Services Task Force; HSgFOBT, high-sensitivity guaiac fecal occult blood testing; FIT, fecal immunochemical testing; OC, colonoscopy; CTC, colonography; FS, flexible sigmoidoscopy

Society	Age (years)	Screening tools with recommended interval	Study references
USPSTF	50-75 (average risk) [19]	HSgFOBT (annually or biennially)	Zauber et al. [20]
		FOBt every two-three years plus FS every five years	Zauber et al. [20]
		OC (every 10 years)	Zauber et al. [20], Knudson et al. [21]
		CTC (every five years)	Knudson et al. [21]
		FS with FIT (FS every 10 years plus FIT annually)	Knudson et al. [21]
		FIT with OC done every 10 years	Knudson et al. [21]
		FIT (annually)	Knudson et al. [21]

Computed tomography colonography

First Look at Its Origin to Present

In 1994, CT colonography (virtual colonoscopy) was presented as a new imaging tool for detecting colorectal polyps and cancer [22]. VC, or CTC, is a procedure that combines CT scanning with image reconstruction using 2D and 3D visualization modalities to view the full colorectum [23]. CTC has evolved through time to serve as a whole colonic examination approach in symptomatic patients suspected of having colonic pathology, as well as a possible screening technique for tiny polyps and early cancer [24]. Various imaging methods include preliminary 3D review and 2D matching for troubleshooting, and diagnosis using computer aid [25-27]. Small polyps were more difficult to visualize with the older technology that used a spiraling CT scanner as it had drawbacks with resolution [28]. A need for crisper resolution of the images was met using the modern multidetector CT scanner that was as swift as holding one's breath, which eliminated the problem of image resolution [28,29]. Many components of this technique are being researched, including software that aids in lesion diagnosis, picture reconstruction enhancements, and stool labeling [29-31]. The last method involves ingesting contrast material over days or hours, and the software digitally removes remaining solid and fluid fecal material from the recorded images, resulting in a "virtually clean" mucosal surface that eliminates the necessity for stool cleansing prior to testing [32,33].

CTC Technique

A day before the CTC procedure, bowel catharsis is performed in which patients take a standard low-volume bowel preparation with either magnesium citrate and bisacodyl or a 2-L polyethylene glycol bowel preparation alone [34]. Single doses of 2% barium sulfate and diatrizoate are used for colonic stool and fluid tagging [11]. All patients are made nothing-per-os (NPO) after midnight before testing [23]. Oral contrast tagging is used in addition to bowel catharsis to detect CTC [35]. The colon is distended with automated low-pressure carbon dioxide supply via the rectum or end colostomy. A diagnostic, IV contrast-enhanced CTC series of the abdomen and pelvis is conducted in the supine position after an unenhanced, low-dose prone, and decubitus series for CTC. Due to minimal intestinal distention, imaging in two positions reduces the risk of insufficient visibility [35]. Also, CTC is performed first in most cases, followed by OC [23]. The CTC evaluation of the entire colorectum is performed on dedicated workstations using 2D and 3D endoluminal imaging. All polyps seen are identified by size, morphology, and location [23].

Diagnostic Accuracy

According to the meta-analysis by Pickhardt et al., the clubbed sensitivities of CTC and OC for CRC were about 96% and 95%, respectively [36]. In conclusion, point estimates of CTC and OC sensitivity for invasive cancer have been presented by this systematic review and meta-analysis. Not only did the pooled sensitivity of CTC for CRC look nearly identical to that of OC, but it was also maintained despite considerable heterogeneity in technique, which is noteworthy in terms of CTC’s representativeness and widespread use. The presence of a significant variation in the data for OC, on the other hand, shows that more research into the test's performance is needed [36]. CTC had a sensitivity of 96.1% for CRC (398 of 414; 95% CI 93.8%, 97.7%). There was no heterogeneity detected (*I*^2^ = 0%). When both cathartic and tagging agents were used in the bowel preparation, no cancers were missed during CTC. The sensitivity of OC for CRC was 94.7%, according to a subset of 25 studies involving 9223 patients (178 of 188; 95% CI 90.4%, 97.2%). There was a reasonable degree of heterogeneity (*I*^2^ = 50%) [36].

Mulhall et al. conducted studies on 6393 patients in 33 trials [37]. The sensitivity of CTC was variable, but it improved as polyp size increased (48%; 95% CI 25%-70%, for polyps <6 mm; for polyps 6-9 mm, sensitivity at 70%, CI 55%-84%; 85%, CI 79%-91%, for polyps >9 mm). CTC scanner characteristics such as collimation width, detector type, and imaging mode explained some of this heterogeneity. Specificity, on the other hand, was consistent (92%, CI 89%-96%] for polyps <6 mm; 93%, 95% CI 91%-95%, for polyps 6-9 mm; 97%, CI 96%-97%, for polyps >9 mm). In the same study, Mulhall et al. noted that the sensitivity of CTC per patient ranged from 21% to 96% [37]. For CTC, the group formed sensitivity was 70% (95% CI 53%-87% ). As the size of the polyp grew larger, so did the sensitivity: polyps <6 mm had a detection rate of 48% (CI 25%-70%; range, 14%-86%), polyps 6-9 mm had a detection rate of 70% (CI 55%-84%; range, 30%-95%), and polyps >9 mm had a detection rate of 85% (CI 79%-91%; range, 48%-100%) [37]. All those analyses were statistically variable (P < 0.001), with between-study heterogeneity accounting for the majority of the variance. The *I*^2^ statistic for polyps <6 mm was 96.7%, 93.1% for polyps 6-9 mm, and 85.2% for polyps >9 mm. They discovered numerous possible sources of heterogeneity. Firstly, trials with smaller collimation slices appeared to have higher sensitivity [37]. A meta-regression of data from 19 investigations found that every 1-mm increase in the collimation width reduced sensitivity by 4.9% (CI 0.8%-7.1%) [37]. Secondly, the reported overall sensitivity in the seven investigations that used multidetector scanners was uniformly high (95%; CI 92%-99%; *I*^2^ = 40%; P > 0.2) [37]. This sensitivity was greater than the average sensitivity reported in nine investigations using a single-detector scanner (82%; CI 76%-92%), while the latter results were diverse (*I*^2^ = 87.1%; P 0.001). The sensitivity of the 10 studies that used 2D imaging with confirmation by 3D imaging only when necessary (*I*^2^ = 87.5% ; P = 0.02) was 81.9% (CI 71%-91%) (*I*^2^ = 87.5%; P = 0.02), while the six studies that used conventional 2D imaging and accompanying 3D imaging had a pooled sensitivity of 91% (CI 83%-99%; *I*^2^ = 53.1%). There was no additional cause of variability after looking at the year of publication, kind of scanner hardware or software used, the width of the reconstruction interval, contrast use (bowel, parenteral, or none), and patient factors (age, sex, and high or average risk) [37]. When the Spearman statistic was calculated, and receiver-operating characteristic curves were created, we observed no evidence of a cutoff effect between sensitivity and specificity. Per-patient specificity was more stable across polyp diameters than the wide range of sensitivities reported [37]. On the basis of data from 14 trials, CTC was 86% specific (CI 84%-88%; *I*^2^ = 92.6%; P = 0.001). As the size of the polyps grew larger, the specificity improved, and the results were consistent across strata. Only four trials revealed specificity for polyps <6 mm, and the combined specificity was 91% (CI 89%-95%; *I*^2^ = 47.1%; P = 0.15) [37]. Specificity was 93% (CI 91%-95%; *I*^2^ = 50%; P = 0.07) for polyps 6-9 mm in size (six studies), and climbed to 97% (CI 96%-97%; *I*^2^ = 41.8%; P > 0.2) for polyps >9 mm (15 studies). Mulhall et al. finally concluded that although CTC is quite specific, there is a wide range of reported sensitivities. Patient or scanner features do not adequately explain this variation, but collimation, scanner type, and imaging mode account for some of them. This variation raises questions about performance consistency and technical unpredictability. Before CTC may be recommended for widespread colorectal cancer screening, several concerns must be addressed [37].

Sato et al. explained that CTC correctly detected 86 of 87 central colon tumors [38]. With the help of CTC and the removal of one minor lesion, they were able to locate all (87/87) colon tumors. As a result, they believe that clipping should be explored to diagnose tiny tumors (less than 10 mm in diameter). CTC produced reconstructed pictures of the primary colon cancer's feeding artery; the feeding artery information received by CTC aided in precise lymph node removal. Although double-contrast barium enema (DCBE) is commonly used to locate colon lesions, CTC outperforms DCBE in key ways [38]. To begin, CTC shows the affected colonic segment, the extent of tumor expansion, its link to neighboring organs, and vascular systems [38]. In their study, Sato et al. also saw that the left colic artery (LCA) originated autonomously from the sigmoid artery in 41% of cases, the LCA and sigmoid artery shared a trunk in 45%, and the LCA did not exist in 5% of cases. When doing laparoscopic surgery, these vascular variances cannot be overlooked [38]. When determining the extent of lymph node dissection, knowing the connection of the lesion to the feeding artery is extremely useful. Because they could forecast the distance to the branch by CTC using a reconstructed picture of the feeding artery, they could finish the lymphadenectomy around the inferior mesenteric artery while conserving the LCA in some cases. Furthermore, CTC can be conducted concurrently with the preoperative CT scan without exposing patients to additional X-rays, and patients do not require the additional purgatives required for DCBE [38]. The pathological T stage was substantially linked with the deformity categorization (P < 0.001, Kruskal-Wallis nonparametric testing).

According to a recent randomized experiment conducted by Halligan et al., patients assigned to CTC had a much greater detection rate of CRC and significant polyps than those assigned to DCBE [39]. Their study readings resonate with those by Horie et al. [40], who established a threshold for stage T3 or T4 based on circumferential tumor extent; the criteria for T3 or T4 staging is 50% as measured by CTC [38]. It has been found that when the diameter of lesions increases, the detection rate of coexisting polyps increases [41]. An OC was done right before CTC in most cases [38]. A polyp greater than 5 mm in size discovered during OC is routinely removed endoscopically before CTC [42,43]. When CTC was conducted in this study, there were only 16 coexisting polyps left; with such a limited number of polyps, they could not assess CTC's capacity to detect coexisting polyps; further research into this element of CTC is required [38].

In Halligan et al.'s study, 3838 individuals were assigned to one of two groups: barium enema (BE; n=2553) and CTC (n=1285) with 2527 patients randomized to BE and 1277 patients assigned to CTC for analysis [39]. Patients assigned to CTC had a significantly greater rate of CRC or large polyps’ detection than those assigned to BE (93 [7.3%] of 1277 vs. 141 [5.6%] of 2527, relative risk 1.31; 95% CI 1.01-1.68; P = 0.0390). BE missed 12 of 85 colorectal tumors, while CTC missed 3 of 45 [39]. Due to a greater polyp identification rate, the rate of extracolonic inquiry was higher after CTC than after BE (283 [23.5%] of 1206 CTC patients had immediate study vs. 422 [18.3%] of 2300 BE patients; P = 0.0003). Serious side effects were uncommon. Halligan et al. finally concluded in their study that the CTC test is more sensitive than the BE test. Their findings show that CTC should be the radiological test of choice for people who have symptoms that could indicate CRC.

In a study by Porté et al., CTC properly indicated anastomotic recurrence in 18 of 19 patients (95%) [44]. Their study shows that CTC had a sensitivity of 95% (95% CI 62-100) and a specificity of 100% (95% CI 75-100) for recognizing anastomotic recurrence in a total of 880 patients under seven investigations. There was no statistical heterogeneity found (*I*^2^ = 0%). They calculated that using CTC as a single test instead of OC and routine CT for surveillance might save an annual cohort of UK patients €20,785,232 (£14,803,404). Porté et al. finally concluded that CTC looks to be more cost-effective than OC. These results should be weighed against the limitations of small patient populations and substantial clinical heterogeneity across trials [44].

In a detailed study done by Singh et al., CTC revealed 40 lesions, including four proximal synchronous lesions [24]. CTC missed one carpet, i.e., flat lesion in the rectum, which was discovered later on traditional OC (confirmed on surgical correlation). The carpet lesion was overlooked due to residual fluid in the rectum, which was discovered retrospectively when CTC was studied. CTC had 97.56% and 100% sensitivity and specificity, respectively, in detecting lesions [24]. The positive predictive value (PPV) and negative predictive value (NPV) were 100% and 93.75%, respectively. Conventional OC had 92.68% and 100% sensitivity and specificity, respectively, in detecting lesions. The PPV and NPV were 100% and 83.3%, respectively. The difference in sensitivity and specificity between CTC and conventional OC had a P-value of 0.305 (not significant) [24]. CTC had 100% sensitivity and specificity for proper 'T' staging, with a PPV and NPV of 77.77% and 100%, respectively, with the accuracy of the diagnosis of 91%. For 22 lesions, a surgical correlation could be found. Fourteen out of 15 T2 lesions were accurately staged at CTC, whereas one was overstaged as T3 due to pericolonic stranding, which was later determined to be attributable to fibrosis on histological investigation. Six out of seven T3 lesions were accurately staged at CTC, whereas one was overstated as T4 due to a loss of fat plane, but there was no invasion of a neighboring organ per-operatively. For accurate 'N' staging, sensitivity and specificity were 100% and 66.67%, respectively, and the PPV and NPV were 88.89% and 100%, respectively, with a diagnostic accuracy of 90.9% [24]. On CTC, malignant lymph nodes were reported with 18 lesions, but histopathology revealed only 16 lesions associated with malignant lymph nodes. Malignant lymph nodes reported with two lesions were reactive [24]. Singh et al. finally concluded from their study that CTC offers greater sensitivity for detecting colorectal cancer than a traditional OC because of its ability to detect anomalies close to the obstructive lesion, as well as appropriate segmental localization and staging. However, significant drawbacks of CTC included difficulty in detection of flat lesions and absence of information concerning hyperemia and superficial mucosal erosion, where traditional OC scored over CTC.

Neri et al. also found that CTC is superior to conventional OC in identifying colonic masses, completeness of colonic examination, and detailed description of the segmental site of the carcinoma in a comparative analysis [45]. In a study by Kumar and Cash, one point of contention was whether CTC could be used to detect tiny polyps measuring 6-9 mm in diameter [46]. The results of a randomized trial of CTC versus OC for screening in 8844 participants were published by Stoop and colleagues (the Colonoscopy or Colonography for Screening [COCOS] trial) [47]. Given the higher enrollment in the CTC group and additional control yield in the OC group, it was determined that CTC and OC had equal yields for diagnosing advanced neoplasia in this experiment [46]. Only CTC participants with lesions with a size >10 mm were referred for OC in the COCOS trial, and those with lesions between 6- and 9-mm size were suggested for surveillance CTC [46]. Only CTC participants with lesions more prominent than 10-mm size were recommended for OC in the COCOS trial, while those with 6- to 9-mm lesions were advised to undergo surveillance CTC [48].

An extensive study conducted by Weinberg et al. showed that CTC gave a sensitivity for patients with polyps less than 6 mm of 44.0% (95% CI 30.2-57.8) and a specificity of 93.4% (95% CI 89.7-97.0) [23]. CTC for polyps less than 10 mm had a sensitivity of 76.9% (95% CI 54.0-99.8) and specificity of 89.0% (95% CI 84.8-93.1). CTC and OC were completed by 231 people. This smaller sample size was adequate to answer the primary and secondary study issues due to higher than expected CTC and OC performance. All participants had Stage 0-III because distant metastatic disease at diagnosis was an exclusion criterion. Stage III illness affected less than half of the patients (48.1%). OC found polyps of any size or histology in 116 of 231 (50.2%) participants. Polyps were found in 50 of 231 (21.6%) and 13 (5.6%) patients with lesions with a size less than 6 and 10 mm, respectively. A total of 36 of 231 (15.6%) patients had conventional and serrated polyps/adenomas of less than 6 mm, whereas 10 of 231 (4.3%) had conventional and serrated polyps/adenomas of size bigger than 10 mm. There were no intraluminal tumors found. They concluded that when compared to extra-luminal recurrences detected by standard CT, intra-luminal and anastomotic recurrences detectable by OC are rare [23]. As a result, if the clinical performance of CTC were satisfactory, it would be preferable to use it instead of higher risk OC.

de Haan et al.'s study shows that CTC yield is observer dependent, much like any other imaging technology [49]. The rate of detection and PPV of CTC are significantly higher at centers with experienced radiologists (>1000 examinations) and centers with more than 175 cases per radiologist per year, according to the analysis made previously of data collected from the English Bowel Cancer Screening Program (BCSP) [50]. This outcome is consistent with the findings of a Dutch study in which six physicians and three radiologists underwent systematic training [51]. The average sensitivity for detecting lesions 6 mm or bigger in size increased from 76% in the first 50 CTC exams to 91% in the fourth set [49]. The number of CTC examinations required for sufficient sensitivity was estimated to be 164, implying that adequate training necessitates at least 164 training cases [49]. Pooler et al. found a much more consistent performance among radiologists experienced with CTC for polyp detection when compared to published alteration among gastroenterologists at OC [49,52].

Extracolonic Detection

Screening for CRC with CTC has the potential to be a novel radiologic approach [53]. Nevertheless, before that, CTC screening requires a satisfactory answer to how to best deal with extracolonic discoveries [6]. Unlike other colorectal screening methods such as OC, FS, and BE research, CTC allows for visualizing organs beyond the colon [53]. The advantage of CTC is that it can detect asymptomatic malignant disease or surgical issues, reducing morbidity and mortality. However, its limitation may reveal several insubstantial findings, resulting in more expensive diagnostic tests and an increase in morbidity [53]. As a result, Hara et al. stated that "further workup of extracolonic CTC findings was very uncommon but often worthwhile when conducted for highly critical lesions" [53]. Extracolonic structure evaluation during CTC has definite limitations in terms of solid organs [53]. However, it can aid in detecting severe diseases without significantly raising the cost per patient. Weinburg et al. in their extensive study, comparing CT colonography versus colonoscopy for colorectal cancer surveillance after surgery, found in two scenarios that CTC detected extramural peri-anastomotic recurrence (0.9%) [23]. In addition, CT imaging revealed new extracolonic illness in 11 individuals, which was compatible with metastatic CRC.

In a study by Hara et al., highly significant extracolonic abnormalities were found in 31% of patients, prompting 18% of patients to have additional testing using modalities such as intravenous pyelography in 1, ultrasonography in 10, and CT in 13 [53]. Incidental pathological findings were seen on CTC. Additional imaging was performed on two patients with moderate or low significance. According to this study, the workup for extracolonic discoveries cost a total of $7324 (average of an incremental $28 per testing). At CTC, three extracolonic cancers were missed. Almost 117 of 151 extracolonic findings in this study were analyzed by the physician and deemed to be of low (68 of 151) or moderate (49 of 151) clinical relevance as they were benign and assumed not to require any surgical intervention. CTC did not result in needless and costly follow-up exams for these results of dubious clinical significance, given the modest radiation dosage (70 mA) and non-enhanced technique [53]. Even though 79 subjects with low to moderate significant results had to undergo further imaging to confirm the presence of renal cysts, the other 77 patients did not. Costs may be higher in institutions that take a more active approach to investigate even small inadvertent results. Because of the CTC findings, the therapy of one patient with a somewhat important finding was altered. This patient had a history of viral hepatitis, and a CTC revealed previously undetected cirrhosis, prompting the decision to monitor the condition using liver enzyme testing. Extracolonic observations were classified as highly important in a small number of patients (30 [11%] out of 264) [53]. Eighteen of those patients had further imaging, which revealed a similar number of patients with malignant findings (n = 5), benign findings (n = 9), and indeterminate results (n = 4). For the remaining 12 patients with previously identified ailments (n = 2), it was advised to have follow-up imaging done at a later time (n = 3) and did not have any subsequent workup noted (n = 10). As a result, it appears that workup for highly suspicious lesions found on unenhanced CTC is uncommon, but when it is done, it is usually effective [53]. Further imaging due to benign disease was done in only a minute percentage of patients with high-, moderate-, or low-importance lesions (11 [4%] of 264), which is critical to note.

Patient Acceptability, Practicability, and Safety Concerns

Zhu et al. showed that CTC had a higher patient participation rate than OC, although the difference was not statistically significant [54]. The rate of non-participation, on the other hand, was statistically significant. Because of higher participation rates, CTC CRC screening is more cost-effective than OC screening [6]. CTC was the preferred test for willingness-to-pay thresholds of €3200 per quality-adjusted life-year (QALY) gained and higher, which is lower than the Dutch willingness-to-pay barrier of €20,000 [6].

The screening population appeared to be more likely to participate in the CTC with minimal or no cathartic preparation [54]. According to statistical evidence, more large randomized controlled trials (RCTs) will be required in the future [54]. However, because the CTC has been validated for CRC screening, the decision on which method to utilize should be made in collaboration with patients, taking into account their preferences as studied by Duarte et al. [18]. Because of the increased participation rate, CTC can now screen for CRC in asymptomatic people, making it an additional screening method [18]. The study looked at the detection rates of advanced colorectal neoplasia (ACN) in 2357 patients who received CTC and 1524 patients who underwent OC. ACN was found in 135 (5.7%) of CTC patients and 130 (8.5%) OC patients. The absolute risk difference between the two process types in the ACN detection rate was 0.02 in favor of OC (with a 95% CI of 0.04 to 0.00).

In a promising study by Pooler et al., "non-invasiveness" (68.0%), "no need of anesthesia" (63.1%), "driving back home with no issues" (49.2%), "prevention of hazards common with OC" (46.9%), and "find lesions outside the colon" (46.9%) were the main reasons for choosing CTC for screening (43.3%) [34]. Only 7.2% of patients reported pain during the CTC examination, and only 2.5% reported more than moderate discomfort. A total of 77.1% of 441 patients who had CTC and optical OC chose CTC, whereas 13.8% preferred optical OC. If CTC had not been available, 29.6% of all patients said they would not have had an optical OC test. A total of 92.9% of patients said their overall CTC experience was "great" or "good," and 93.0% said they would use CTC for their following assessment. These findings imply that if CTC is widely available, it can boost commitment to CRC screening standards. They concluded that the patients' experiences with CTC as a primary CRC screening test were overwhelmingly favorable [34].

According to Porté et al., CTC offers single-test luminal, serosal, and extended colonic assessment, compared to OC for CRC surveillance [44]. Standard surveillance techniques could be replaced with CTC, saving money. In the case of CTC, there are two areas where over-detection may be relevant: the diagnosis of polyps that would not have progressed to cancer if left alone and the recognition of extracolonic lesions that would not have shown clinically if left alone [55]. When compared to optical OC, the use of CTC for CRC testing provides effective screening, patient-centered benefits, and cheaper costs, and may be especially appealing to the currently unscreened population with commercial health insurance [56]. Sawhney et al. showed that 50% or less cost would be incurred with the usage of CTC per screening year than optical OC if the availability of the technology expands to match the rising demand [56]. In the BCSP study, a national assessment of CTC indicated that 10% of radiologists doing CTC tests had received no professional training, and one-third of radiologists evaluating the pictures were unskilled [57].

Lara et al. demonstrated that even after a mucosal biopsy during an incomplete OC, same-day CTC is safe, in their recently presented retrospective review of 6260 OC patients, of whom 198 (3.1%) received same-day CTC due to incomplete OC [58]. Thirty-four (17%) of these individuals had had a cumulative of 72 polypectomies in the 24 hours leading up to same-day CTC. During short- and long-term follow-ups of all patients undergoing same-day CTC in this trial, no problems or perforations were reported. Most polyps removed prior to same-day CTC in this study were minor or insignificant, with only 5 (7%) polyps bigger than 10 mm excised prior to OC [58]. Approximately 75% of polypectomies were performed on the left colon. More information on the safety of same-day CTC following incomplete OC with polypectomy is needed, even while this information is reassuring [46].

A detailed study done by Kim et al. showed that although the numbers of polypectomies and sequelae were significantly lower in the CTC group, both primary CTC and OC screening techniques resulted in equal detection rates for advanced neoplasia [35]. This data supports CTC used as a preliminary screening test prior to treatment OC. Cancer risk connected with false positives, difficulties spotting flat adenomatous lesions, and the difficulty to remove polyps or worrisome biopsy lesions during the process, prompting referral to diagnostic OC are all disadvantages of CTC [55]. There were no problems linked with CTC in any patients [38]. CTC also produced rebuilt pictures of the feeding artery in primary colon tumors, which aided in safe and exact lymph node dissection [38]. Following OC, no adverse events requiring extended observation or hospitalization were noted [23]. One individual was given antibiotics and was kept under close supervision for a suspected perforation after the CTC [23]. There was no need for surgery in this case.

In order to prepare the colon for CTC, the bowel is inflated with either room air or CO_2_, which increases the risk of perforation. The frequency of perforation is estimated differently by different people [55]. Nevertheless, perforation is not always the danger that needs to be linked to using CTC [59]. Also, complications during examinations are rare with CTC [60]. The perforation rate for 18 patients out of 50,860 was recorded as 0.035%, which also takes into account asymptomatic extracolonic gas that did not require any intervention, while the symptomatic perforation rate was much less being 0.015% for patients undergoing CTC either for screening purposes or because of symptoms [60-63]. The symptomatic perforation rate ranging from 0.2% to 0.02% was recorded exclusively when using CTC for primary OC screening [60-63]. Fifteen out of 18 participants who had a perforation due to CTC had it due to manual insufflation, which is no longer considered a cutting-edge methodology [49]. A mere seven asymptomatic perforations occurred out of 40,121 screenings and diagnostic CTCs performed in a large-scale Italian research study [64]. One patient had a prior OC and a biopsy two weeks before undergoing CTC [64]. de Haan et al. did not record any symptomatic bowel perforations, but patients with a history of inflammatory bowel disease and diverticulitis in acute-setting CTC were not indicated [49]. Also, the risk of perforation was favorably high in patients who underwent a recent OC with biopsy or polypectomy. The complications occurring after OC as a follow-up to positive CTC findings should also be considered when drawing a CTC contrast with other screening methods [49]. Screening using OC and CTC in a randomized trial showed post-polypectomy bleeding (0.3% for CTC and 0.2% for OC), but showed no perforations [47].

For paired CTC scans, the absolute lifetime cancer risk using an average radiation dose and current scanner techniques was estimated to be 0.14% for a 50-year-old and about half of that for a 70-year-old [55]. Particularly with newer technologies available, the dangers of radiation exposure are probably overstated [49]. Patient doses are substantially lower these days and are likely to continue to fall. The risk, however, is genuine, and screening age groups and intervals should reflect this [55]. Table 2 shows various studies conducted using CTC as a diagnostic modality [55]. It is questionable if episodic radiation exposure from radiographic tests causes a substantial increase in long-term cancer risk [59].

**Table 2:** Population study data for CTC CRC, colorectal cancer; CTC, computed tomographic colonography; OC, colonoscopy; ACN, advanced colorectal neoplasia; QALY, quality-adjusted life-year; NPV, negative predictive value; PPV, positive predictive value

Author, year	Study type	Sample population	Population age (years), country	Diagnostic modality	Results	Conclusion/comments
van der Meulen et al. (2018) [6]	Randomized controlled screening trial	Participation for colonoscopy (OC): 21.5% (1276 of 5924 invitees). CT colonography: 33.6% (982 of 2920 invitees)	Regional municipal administration registries; 50-75 years; Amsterdam and Rotterdam	CTC vs. OC	In screening methods with one or two lifetime tests, OC was more cost-effective, whereas CTC was more cost-effective in strategies with more lifetime screenings.	CRC screening using CTC is more cost-effective than OC screening because of the more excellent participation rates.
Duarte et al. (2018) [18]	Systematic review	2333 of 8104 (29%) patients underwent the CTC, and almost 20% of patients out of 7310, which is a total of 1,486, underwent OC.	Asymptomatic patients ≥50 years	CRC compared to OC	CTC has been demonstrated to be ineffective in detecting ACN.	In asymptomatic patients, CTC can be used to screen for CRC. However, because CTC is less effective at identifying ACN, it should not be used to completely replace OC as it is still considered the gold standard technique. So patients should be informed of OC's superiority in ACN detection.
Weinberg et al. (2017) [23]	Systematic review	231	Resected Stage 0-III CRC from 5 tertiary care academic centers	CTC + OC	Sensitivity of 44.0% for polyps less than 6 mm and a specificity of 93.4 for CTC	CTC was inferior to OC in detecting patients with polyps less than 6 mm in a CRC surveillance cohort one year after resection.
Sato et al. (2016) [38]	-	86	Colon cancer patients	CTC	Prior to surgery, CTC accurately detected all 87 primary colon tumors. CTC did not cause any issues in any of the patients.	CTC appears to be a realistic and helpful preoperative examination technique for colon cancer treatment.
Singh et al. (2015) [24]	-	50	Clinical symptoms of colonic pathology	CTC + OC	CT colonography detected two synchronous lesions proximal to the occlusive mass and one synchronous lesion proximal to the anastomotic location missed by conventional colonoscopy.	CT colonography offers greater sensitivity for detecting CRC than traditional colonoscopy. The drawback is flat adenomas; hyperemia cannot be as well detected as in OC.
Pooler et al. (2012) [34]	-	1417	Multicenter	CTC + OC	CTC was rated as having a very high level of satisfaction by participants, and those who had used both modalities said that CTC was preferred over optical OC.	Finally, the findings concluded that patients' perceptions of CTC as a primary CRC screening test were extraordinarily positive. It has the potential to elevate compliance to global CRC screening guidelines.
Hanly et al. (2012) [55]		16 studies	United States, Canada, France, Italy, and the United Kingdom	CTC	CTC was more cost-friendly than flexible sigmoidoscopy and fecal occult blood testing. CTC expenses, screening uptake, polyp referral threshold, and extracolonic discoveries were the factors that had the most significant impact on cost-effectiveness.	The literature on the cost-effectiveness of CTC screening is mixed, owing to changes in comparators and parameter values between studies.
Pickhardt et al. (2011) [36]	Systematic review	11,151	Colorectal cancer was prevalent in 3.6% of patients (414 cancers)	CTC vs. OC	Data shows that, assuming a sufficient level of specificity, primary CTC may be more suitable than OC for the initial assessment of suspected CRC, given the relatively low prevalence of colorectal cancer, even among symptomatic groups.	When both cathartic and tagging agents are used in bowel preparation, CTC is extremely sensitive for CRC.

Future implications

Future research should pinpoint and stress developing a more detailed and comprehensive model of CTC characteristics to guide efforts to improve this relatively new technology and optimize its potential for enhancing testing compliance in both cancer screening contexts [65]. Table 3 demonstrates some noteworthy differences between CTC and OC [65]. Now that the CTC technique has emerged and reporting excellence is possible, CTC screening studies should focus on establishing more rigorous training and quality assurance programs, as well as the recognition of quantifiable key performance indicators (KPIs) that can anticipate clinically relevant outcomes in the coming years [66]. More research into the natural history and pathophysiology of CRC will aid in determining the best adenoma size parameters for referral to OC and the durations of CTC screening intervals. Real-world data, current management options, detailed models of colorectal polyp biology, and extracolonic outcomes should be used to improve existing estimates of cost-effectiveness. To help with this, screening CTC radiologists must follow the same rules as their colleagues in screening OC, such as disclosing both symptoms and screening CTC in routine practice [66]. CTC can raise colon cancer screening rates due to its relative safety, low cost, and patient acceptance [67]. Due to its extensive fluctuating sensitivity, difficulty sampling polyps for histological study, and lack of curative capacities, its function in widespread CRC screening is controversial [67]. Whether the continuous use of CTC will increase or decrease the frequency of referrals for OC or whether the process will shift from CRC screening to treatment measures (e.g., polypectomy) is unknown [68]. Communication and coordination between gastroenterology and diagnostic imaging will be necessary.

**Table 3:** A comparison between CT colonography and colonoscopy

Features	Colonoscopy	CT colonography	Study reference
Image format	Real-time format	2D and 3D image format; high definition resolution	von Wagner et al. [65]; Weinberg et al. [23]
Patient logistics			
Patient participation/adherence	Low	High	Pooler et al. [34]
Postprocedural patient status	Brief observation	No hospitalization required	Weinberg et al. [23]
Drive back themselves	No	Yes	Pooler et al. [34]
Patient's choice	13.8%	77.1%	Pooler et al. [34]
Cost-effective	No	Yes	Sawhney et al. [56]; Porté et al. [44]; van der Meulen et al. [6]
Problems in viability and practice			
Prerequisite - anesthesia	Sedating the patient required	No sedation	Weinberg et al. [23]
Bowel prepping	Bowel cleansing prior to starting	Bowel cleansing required	Weinberg et al. [23]
Total duration	2 hours	30 min	von Wagner et al. [65]
Complications			
Uneasiness	Mild	Mild	Weinberg et al. [23]
Bleeding	1 in 300	No	von Wagner et al. [65]
Perforation	1 in 800	1 in 3300	Hanly et al. [55]
Radiation dose	No	0.14% (for age 50)	Hanly et al. [55]
Extracolonic detection	No	Yes	von Wagner et al. [65]; Hara et al. [53]; Weinberg et al. [23]
Lesion detection accuracy			
Detection of flat adenomas	Better	Failed	Singh et al. [24]
Evidence of hyperemia	Yes	No	Singh et al. [24]
The imagery of feeding artery	No	Yes	Sato et al. [38]
Patient participation rate and adherence	Low	High	van der Meulen et al. [6]; Duarte et al. [18]; Pooler et al. [34]; Zhu et al. [54]
Test sensitivity	95%	96%	Pickhardt et al. [36]
Sensitivity	93.75%	97.56%	Singh et al. [24]
Specificity	92.68%	100%	Singh et al. [24]

Limitation

One limitation of this review is that we primarily compared CTC to OC in most cases when there are several other diagnostic and screening modalities present.

## Conclusions

CTC proves to be an effective surveillance modality for CRC, as evidenced by this literature review. Since the advantages of CTC outweigh the limitations, CTC needs to be widely incorporated and advocated into the daily practice of screening CRC in the future due to patient ease factor, its efficiency to detect cancerous lesions and its perks of being financially feasible for the patients, thus enhancing its practicability. Having looked at the high sensitivity and specificity as compared to OC and high NPV of this test, despite the minute flaws, improvement in CTC techniques has led to its integration in the USPSTF screening guidelines for colon cancer. Keeping that in mind, CTC has a significant pitfall due to overdetection of non-cancerous lesions and phantom polyps, which can be either colonic or extracolonic, and could be due to the lack of interpretation skills when using CTC. To add to the available resources, this article would prove an essential tool in highlighting the importance of CTC for colon cancer screening. Also, more thorough studies need to be done regarding the bowel preparation and other limitations for it to make a meaningful asset rather than a substitutive modality for CRC.
